# User fee exemption policies in Mali: sustainability jeopardized by the malfunctioning of the health system

**DOI:** 10.1186/1472-6963-15-S3-S8

**Published:** 2015-11-06

**Authors:** Laurence Touré

**Affiliations:** 1 Association de recherche Miseli, Mali

## Abstract

In Mali, where rates of attendance at healthcare facilities remain far below what is needed, three user fee exemption policies were instituted to promote access to care. These related to HIV/AIDS treatment, as of 2004, caesarean sections, since 2005, and treatment of malaria in children under five and pregnant women, since 2007. Our qualitative study compared these three policies, looking at their implementation provisions, functioning and outcomes. In each healthcare facility, we analysed documentation and carried out three months of on-site observations. We also conducted a total of 254 formal and informal interviews with health personnel and patients.

While these exemptions substantially improved users' access to care, their implementation revealed deep dysfunctions in the health system that undermined them all, regardless of the policy studied. These policies provoked resistance among health professionals that manifested in their practices and revealed, in particular, the profit-generation logic within which they operate today. These dysfunctions reflect the State's incapacity to exercise its regulatory role and to establish policies that are aligned with the way the health system really works.

## Introduction

The conditions governing access to basic facilities are a source of ever-increasing inequality in a number of sub-Saharan countries and seriously jeopardize achievement of the Millennium Development Goals by 2015 [1]. Under pressure from international bodies, and mindful of the need to correct the negative impact of the cost recovery system, states have tried to improve access to healthcare [2]. This is the background against which policies of total or partial fee exemptions have been adopted, in partial divergence from the previous cost recovery policy. Three user fee exemptions have been introduced in Mali, where attendance at health centres falls well below real needs (primary care consultations rose from 0.18 new cases per inhabitant per year in 1998 to 0.35 in 2010). These three exemptions relate to HIV/AIDS treatment, as of 2004, caesarean sections, since 2005, and the treatment of malaria in children under five and pregnant women, since 2007.

Documentation of these policies has been slow, especially in West Africa [2,3]. Studies initially focused on the policies' impacts and encountered certain difficulties in evaluating the effectiveness of complex interventions in their natural context [4,5]. The achievement of anticipated effects (increased visits to healthcare facilities and improved access for the poorest families) has been widely documented through quantitative surveys [6,7]. For example, studies in the Kayes region and Bamako in Mali showed that free caesareans have significantly reduced catastrophic expenditure associated with such treatment, mainly among women living in urban areas [8]. The free availability of anti-malarial drugs for children under five has led to a perceptible rise in the number of visits to health centres, although the increase is smaller than expected [7,9].

Regarding the conditions surrounding implementation of the new policies, a 2011 national conference on fee exemption policies in Bamako [10] reported on some concerns among health professionals, circle councils, and community health associations regarding these free care policies. They were especially concerned about their financial viability and about the impacts of user fee exemptions for malaria treatment on the financial autonomy of community health centres. They also stressed the need to plan and reinforce measures to support these policies. At the conclusion of this national conference, all stakeholders pointed to the need for more knowledge on the dynamics of the implementation process.

After these policies had been in force for a few years, it was important to (a) document and compare the uptake of user fee exemptions policies by those responsible for their implementation (i.e., health workers) and (b) evaluate the policies' impact on the health system and the circular nature of the dynamic. Exemption policies in Africa have had an impact on most health system functions [11], with respect to WHO and universal coverage [12]. However, in the specific context of our study in Mali, we studied essentially the impacts on the health services provision function. We compared different policies in identical contexts (an approach rarely used before now) to better understand the factors underlying their success or failure and particularly what role the common substratum, i.e., the health system and its provision of services, played in the results.

## Methods

This was a qualitative study involving case studies [13]. We began by conducting interviews at the national level and consulting available documentation (service notes, activity reports, etc.) to understand the framework established for each policy, as a foundation for analysing the gap between those provisions and the actual conditions of implementation on the ground (Table 1).

**Table 1:** Number and types of interviews conducted at the national level.

Groups	Number
Managers in the Ministry of Health, at the national and regional levels	16

Managers in the HIV/AIDS program	8

Managers in the malaria program	3

Managers at the Ministry of Health specialized in caesareans	1

Technical and financial partners (United Nations agencies, bilateral cooperation agencies)	16

National NGOs	9

International NGOs in Mali	6

Medical experts	3

Officials from the presidency of the republic	2

Total	64

To study the free care policies related to caesareans and malaria, we selected Bamako and the regions of Kayes and Sikasso. For the policy related to HIV, we focussed only on Bamako.

Bamako was selected because it is an urban environment that is close to decision-making and supply points. In Bamako, we selected the Commune 1 health district because it contains Mali's first CSCOM (community health centre), has a well-recognized CSREF (referral hospital), and is made up of neighbourhoods of very different standings. The Kayes region was selected because the Canadian Council for International Co-operation is involved in maternal health in that region and has conducted quantitative studies that are complementary to our qualitative approach [14]. In that region we selected Kita district because it is also a sentinel site for malaria control, which suggested good conditions for implementation. In the Sikasso region, we selected the Sikasso district because it offered a rather advantageous health setting, with no major structural impediments to the successful implementation of major free care policies. In these three districts of the three regions, we selected three health centres. First, the three referral health centres (CSREFs) were studied, as these are the district referral hospitals. Then, we selected the two most contrasted community health centres (CSCOM) in the districts. The contrast was determined based on their degree of isolation, which has an influence on information, supervision, and drug supply, but also on health worker and community organization dynamics, as well as on the level of use of the CSCOM by the population (Table 2). The management of caesareans was studied in two CSREFs in Bamako and Kita, and at the Sikasso regional hospital, a public institution that enjoys a degree of independent management. We observed a total of 58 cases of caesareans at the three sites.

**Table 2:** The nine sites involved in the study of the three policies.

Study district	Free malaria care policy		Free caesarean policy	Free HIV/AIDS care policy
Sikasso	CSREF Sikasso	District hospital	Regional hospital	N/A

	Urban CSCOM Sanoubougou	Urban, well attended, dynamic team		

	Rural CSCOM Kafana	Rural, isolated, health mutual, well enough attended		

Bamako	CSREF Commune 1, Bamako	District hospital	CSREF Commune 1	CSCOM Commune 2, Bamako, integrated AIDS procedure

	CSCOM Commune 1 Banconi	Good performance, oldest in the country, popular neighbourhood		USAC Commune 1 Bamako; AIDS procedure = associative unit in the public centre

	CSCOM Commune 1 Djelibougou	Recent, well-off neighbourhood		CESAC Associative centre

Kita	CSREF Kita	District hospital	CSREF Kita	N/A

	CSCOM Djidian	Isolated, elderly retired physician, community dynamics		

	CSCOM Badinko	Well located but poorly attended, new physician		

To vary the management status of multiple sites (public, semi-public or private centres, which do not function in the same ways) in a single given context, we studied the management of HIV/AIDS patients in the district of Bamako alone, selecting three sites. The first was the CESAC (advice and counselling centre), an associative-and, as such, private-treatment centre run by ARCAD-SIDA (association for research, communication and home assistance for persons living with HIV/AIDS) and devoted exclusively to the management of HIV/AIDS. The second was the CSREF of Commune 2, a public health centre offering fully integrated management of the disease. The third was the USAC (care, support and counselling unit) of the CSREF of Commune 1. This unit, which specialized in HIV/AIDs, was located in a public health centre but had a certain level of autonomy; it was funded by ARCAD-SIDA with the CSREF's backing.

In each site, the research team carried out a documentary analysis (data produced by the health information service and service notes), three months of on-site observation, and formal and informal interviews with healthcare workers and patients. Our team was made up of a researcher in charge of studying the emergence and implementation of the three policies at the national level and a team of three research assistants supervised by the author of the article; each research assistant was in charge of one of the study sites, for each of the three policies. We conducted in-depth qualitative interviews with different categories of personnel (service chiefs, physicians and residents, nurses or midwives, nurses' aides or matrons) in the services affected by the free care policies in each of the selected health centres. Respondents were selected to ensure that all categories of health workers were represented in our sample so that all possible viewpoints would be included. We also interviewed patients and those accompanying them during our on-site observation periods, whose care we were able to observe before their discharge (Table 3).

**Table 3:** Number and types of interviews conducted in the sites.

Free healthcare policies	Number of interviews with health professionals	Number of interviews with community leaders*	Number of interviews with patients/carers
Caesareans	36		25

Malaria	36	6	37

HIV/AIDS	35		15

Total	107	6	77

* Community leaders are involved only in community health centres (CSCOM) and have no role with regard to caesareans or HIV/AIDS management.

By triangulating information in this way and by long immersion in the field, we were able to record not only interactions between actors, but also the conditions in which patients were managed, above and beyond what health professionals reported about their practices. The data (daily observations recorded in notebooks, interview transcripts) were subjected to thematic analysis (with the construction of a thematic tree) and interpretation. The data were examined and discussed, and data relating to different themes and sites were compared. The research was authorized by the ethics committee of the INRSP (National Institute of Public Health Research: 9-000011/CE-INRSP). The field surveys were conducted between September 2010 and December 2011, and the research findings were presented at a meeting in Bamako in November 2012.

The three policies are presented below. In each case we focus first on the action plan adopted for each and then on their operationalization and the results achieved. Finally, we show how malfunctions within the health system had much the same effect on all three policies, despite the policies' very different functions.

## Results

### The policy of free healthcare for HIV/AIDS treatment

#### National policy background

As defined by law in 2006, free healthcare for HIV/AIDS sufferers entails access to antiretroviral drugs and to contraceptives for men and women; absorption of the cost of consultations, analyses, and screening; and the biological monitoring and treatment of opportunistic diseases. Hence, the policy confers substantial and undisputed rights on a single category of patients.

Unchallenged at the national level, the user fee exemption for HIV/AIDS patients also benefits from considerable financial support from international partners, especially the Global Fund. From 2006 to 2009, internal resources only amounted to between 18% and 26% of the total budget allocated to HIV/AIDS in Mali [15]. Since its introduction, the fee exemption has also benefited from extensive technical support in the form of management of supplies (specialist software, technical assistants, training and logistics), a training policy of unrivalled scope, the establishment of a follow-up system with the resources to match, and a monitoring system. However, this monitoring system was essentially based on quantitative indicators, designed to render accounts to donor agencies, and as such not very useful for capturing the implementation process as required for our study.

"This type of free healthcare has more partners than the others. The partners even provide logistical support for distributing ARVs at the regional level. Effectiveness in combating AIDS is determined at the partner level," (Official, Regional health authority).

Another distinguishing feature of this policy in Mali is that it is largely implemented at the operational level by the associative sector, which is private. The HIV/AIDS treatment programme was set up by the association ARCAD-SIDA at a time when the State confined its involvement to promoting prevention. Thus, the programme to fight the disease was largely developed alongside government structures, around and with the support of community associations specializing in the medical management of the disease. This was something new to Mali. On the strength of donations from the Global Fund, measures were negotiated to support this form of free healthcare. These measures included advantageous conditions for those working in associative structures, adherence clubs, and home visits, as well as salaries for psychosocial counsellors providing comprehensive management and for data entry clerks to support more careful planning; these latter personnel could in fact earn the same or more than physicians working in the public sector.

"In the beginning, our relations with the hospital staff were good, but since the Global Fund came in, the doctors, who often earn less than what is paid to us, the psychosocial counsellors, are frustrated. This translates into a refusal to collaborate. The pharmacist doesn't send us the list of patients lost to follow-up and doesn't send us non-compliant patients." (PLHIV, member of a seropositive patients association).

At the time of our study, the State was heavily involved in fighting the disease; however, associative organizations still managed more than 50% of the patients being treated.

#### The implementation of this policy in healthcare facilities

This free healthcare policy has been a success on the whole, thanks in large measure to both the technical and financial support provided from abroad and the dynamism of the associative sector. As a result, there has been an exponential increase in the number of patients receiving treatment. According to official figures [16], in December 2011, of the 42,000 individuals estimated by USAIDS to be in need of treatment in Mali, 29,237 were taking antiretroviral drugs and being regularly monitored at 100 healthcare sites, which distribute the drugs free of charge.

Compared with the two other conditions studied (malaria and caesareans), and according to the observations and interviews conducted in the different health services during this study, the quality of the system providing patient care, including the public system, is reasonably satisfactory. Our study showed that, in a healthcare system characterized by a general climate of indifference towards patients as individuals and nearly non-existent communication between caregivers and patients, the relations that have developed with people living with AIDS and HIV (PLWHIV) are completely unprecedented and privileged.

"We have to show those suffering from the disease that we are not afraid of them, and show more concern for them socially as individuals. Often, one becomes friends with the person, more so than with other illnesses," (Physician, CSREF).

"With Dr X., I feel as if she's family; she's interested in all her patients' social problems and gives good advice. You feel comfortable with her, you're on the same level. Dr X. is willing to spend the time to calm you down, to reassure you, so that you even end up forgetting that you're ill," (PLWHIV, public system patient).

Our empirical results showed that, in practice, both health workers and patients reported that these improvements took the form of a more individualized approach and more specialized response, imposed by national and international standards but also relatively well internalized: longer consultation periods; explanations given to patients about the different aspects of their illness; real-time listening; and visible efforts to ensure a degree of confidentiality in a setting that militates against it, given the level of responsibility imposed on the caregivers and the cramped premises.

Nevertheless, while demand at the global level had been increasing exponentially, the funding for HIV had fallen off markedly. In Mali, this situation led to narrower targeting of resources, a development that prompted serious frustration within the country and produced cracks in the model system. Beginning in 2008/2009, as needs increased significantly and the worsening financial crisis led to a drop in rich countries' contributions to the "common pot" that was the Global Fund, fewer funds were available at the global level for HIV/AIDS. Difficult negotiations had to be undertaken at the national level, and priority was given to medical care and ARV availability, to the detriment of prevention or community support activities, largely provided by the associative sector.

The comparative approach we adopted in our study revealed the existence of a two-tiered system. The associative sector had managed to sustain the gains it made in terms of quality of services and the free healthcare package by resorting to funding that was parallel to that of the Global Fund (and particularly the financial contribution of international NGOs such as AIDES, etc.). In the public sector, there was a growing tendency for the management of HIV-positive patients to be de-prioritized as these patients were increasingly subjected to the usual public services malfunctions that affect quality of care. Both health workers in the services involved and patients reported long waiting times for consultations, the lack of even a superficial rapport with the prescribing or dispensing doctor, staff turnover, the repeated absence of skilled workers, and faulty equipment. Also in the public health system, according to the worried observation of the Cellule Sectorielle de Lutte Contre le Sida (CSLS - Sectoral AIDS Control Cell) with whom we met, there was an appreciable increase in the number of patients lost to follow-up. On top of all this, the scope of free care was gradually shrinking, especially due to the unpredictable availability of reagents that resulted in longer intervals between check-ups. Stock shortages of medicines to prevent or treat opportunistic infections were becoming more common, and community and support services (e.g. support groups, communal meals) were being abandoned.

The Global Fund temporarily withdrew its funding in late 2010 after an investigative mission by the Office of the Inspector General ostensibly identified irregularities in both the public and associative sectors. While this interruption of funding had serious consequences, in a context of political and security tensions (overthrow of the government, occupation of northern Mali by jihadists), it showed the resilience of the HIV/AIDS management system, owing greatly to the part played by community associations and the technical and financial support received. Our interviews with officials in the national HIV/AIDS programme, with international partners, and with workers in the treatment centres showed that, despite not being paid for several months, until the Global Fund resumed funding, association staff carried on dispensing ARVs and operating the follow-up system, without which the activities would have been seriously compromised. Free healthcare during that time was confined to the management of ARVs, which had been maintained thanks to a significant financial contribution by the State. They were managed with the utmost rigour, and every precaution was taken so that stock shortages would not become an issue; in particular, a very effective network was established between community and government management bodies to facilitate a constant exchange of free inputs.

However, according to the same respondents encountered in our study, donors said that such management was proving too costly, since the system was largely funded from outside the country. More than half the patients taking ARVs were being treated in sites run by associations, whose funding (for human resources and operating costs) was almost entirely dependent on the Global Fund.

"Donors feel that health management is very expensive in Mali because most of the work is undertaken by private bodies, with workers being paid four or five times more, not to mention the other costs," (Staff member, international NGO).

The succession of crises that have undermined the fight against HIV/AIDS has persuaded the Malian government and its international partners to consider institutionalizing the HIV/AIDS programme. To generate economies of scale and to avoid escalation of financial dependency, there is talk of promoting integrated management of the various chronic diseases, and "of ending the isolation surrounding AIDS" (according to an official on the Health Ministry's National HIV/AIDS Committee) by gradually incorporating the associative sector into the public one-despite the public sector's de-prioritization of HIV/AIDS management, and at the risk of compromising outcomes. These fears were expressed by respondents in the associative sector as well as in the CSLS, who pointed to the poorer quality of treatment provided in the public sector.

### The free healthcare policy for caesareans

#### National policy background

The free healthcare policy relating to caesareans is a measure of the State's determination to reaffirm its sovereignty in decisions about national healthcare policies vis-à-vis its technical and financial partners [15]. The policy is funded entirely from the national budget. However, in an effort to contain costs, neither the support measures nor the supervision programme contemplated in the preliminary discussions were retained. Nor did this scheme benefit from the technical support of partners; even today, instances of support from international collaborators are few and far between, mainly taking the form of communication campaigns and the funding of a national evaluation.

The policy is one of an on-going series of reforms aimed at improving technical facilities and human resources for maternal health. In the case of caesareans, free treatment applies to the surgical procedure and prior examinations, kits for operations and post-operative treatment, and hospitalization. At the time of our interviews, although aware of the serious problems posed by dystocic pregnancies before free healthcare, staff had reservations about the State's ability to sustain this policy due to the high costs involved. They accepted it, but without enthusiasm, especially since the measure was announced before the technical system was discussed and devised. In fact, its scope was unclear and difficult to pin down, and the initial scheme did not provide for the management of complicated caesareans. The kit for caesareans was based on hospital standards in Bamako, with no consultation or respect for practices in the health centres performing caesareans. To make matters worse, at the operational level, healthcare facilities were compelled to borrow from their own regular stocks to compensate for failures in the supply of inputs for the free caesareans policy from the central level (erratic funding, incomplete kits).

It quickly became obvious that the number of caesareans was growing markedly and steadily. The rate for the country as a whole rose from 0.94% in 2005 to 2.17% in 2008 [17], despite these shortcomings and a deficient referral and evacuation system, which, as our comparative analysis has shown (interviews with personnel, accounts of evacuation conditions for rural patients), penalizes rural areas and women from disadvantaged backgrounds.

"Our greatest difficulty is the more than 100 km distance between our CSCOM and the CSREF. In my two years as medical chief of this CSCOM, the community health association and the mayor's office have not done their share. Because of this, patients have had to pay the ambulance charges themselves, sometimes as much as 37,000F [XOF]," (Medical chief, remote CSCOM, Kita).

#### The implementation of this policy in healthcare facilities

As our study began in 2010, we were unable to observe the management of caesareans before the introduction of the free care policy. However, the accounts of women who underwent caesareans before and after the policy and of health professionals involved in those services revealed that staff physicians often handed the procedure over to other physicians, either from within or outside their centre, rather than performing the procedure with their assistance. The latter were poorly paid (7 500 XOF per month), as they were considered trainees. They were employed for on-call duty but also for day-time service. Staff physicians devoted a portion of their work time to interventions in the private sector, working in the public sector primarily in cases of emergencies (serious complications) or when performing scheduled caesareans on patients with whom "financial arrangements" (fees paid to the gynaecologist by the patient) were made during their pregnancy care. Both staff physicians and trainees also sought to supplement incomes they considered to be low (even non-existent, in the case of trainees) by charging patients unwarranted fees.

The free caesarean policy disrupted this usual way of functioning in the services involved, by increasing workloads and depriving health professionals of these "compensatory" informal payments.

"Before free caesareans, the gynaecologists made up their own kits according to their orders, and they were able to make a profit on those products. Often, the gynaecologist would bring his own products, but would take the money and then use his own products, and this, on top of what we were paid by parents for the act. This free care policy is a loss for gynaecologists, I won't deny it. Even if we take money from the women, it's not like before the free care, as the products [kits] are given free of charge, even though reconstituted kits mean that those accompanying the women pay for the products," (Personnel, Sikasso).

A few years into this policy, local staff came to realize that not only was there an absolute increase in their workload, as more caesareans were being carried out, but also that they were losing out financially.

In fact, the sums reimbursed by the State were not being redistributed among the staff but were being folded into the general revenues of the health centres; at best, some of this income was distributed in annual bonuses, which were seen as insignificant. In addition, the amount paid by the State (XOF 10,000) per procedure was well short of the amount a gynaecologist could have obtained before, informally, for a caesarean.

The difficulties encountered at the national level (erratic funding, incomplete kits) added to the dissatisfaction [17]. There was no hope of securing support measures for this policy, such as incentive payments, as these would involve further expenditure by a government already struggling to cope with the policy's spiralling costs. According to the Finance Department of the Ministry of Health, between 2005 and 2009, the average annual funding rose from XOF 461 million in 2004 to XOF 1,753 billion in 2009, an average annual rate of increase of 40%. A growing discontent was observed among staff physicians, especially those able to work in private clinics, as was the case in Bamako or Sikasso, and the quality of services, already limited, was compromised even further. This undoubtedly explained the increase in puerperal infections and suppurations between 2006 and 2009 [17].

"The facts are unpalatable but they still need stating: it's the junior doctors who perform the majority of caesareans without being supervised by the gynaecologist, who spends all his time on consultations and private work to make more money. This explains why a number of women experience infections and other internal problems after their operations, such as uterine ruptures, etc. We all like earning money, and there's no money in caesareans anymore," (Physician, CSREF, Bamako).

The healthcare facilities often 'trimmed-down' the kits received, as the local professionals generally considered their contents to be too elaborate, excessive, and not always necessary. This conclusion was debatable, as the hospital experts at the central level who determined the kits' contents considered them essential. Occasionally the contents were stretched to create more kits. However, items that were removed and did not go into other kits were neither given to patients nor returned to the pool of non-chargeable items, and were often sold for personal gain.

"The system of free caesareans is chaotic, and there are no proper standards. For example, each centre actually puts its own kits together in any way it pleases. There is a standard content decided by academics, but the fact of the matter is that, if the centre receives 100 kits, it will make them stretch to 150 by using the same items, as some of these are sent in large quantities. And the 50 extra ones come in very handy for the anaesthetists, the gynaecologists and the pharmacy," (Staff member, CSREF, Sikasso).

The rise in caesareans also put extra pressure on equipment, which was often of poor quality, badly managed (electricity bills not paid, waste, theft) and badly maintained (machines not working, new equipment not installed).

"Surgeons have enough equipment to perform caesareans but just don't look after it. Equipment is stolen by workers for their own clinics, and, worse still, things are often thrown into the wash after an operation and, when they come out again, are not put back," (Manager, CSREF, Bamako).

A victim of the policy's success (increase in caesareans) and of the wasting of free inputs, in 2011 the State was faced with a growing deficit for this policy. It reacted by allowing delays to accumulate in the payment of orders placed by the Treasury (basically, kits for caesareans), which left the public pharmacy, the Pharmacie Populaire du Mali (PPM), vulnerable and caused a degree of disruption and interruptions to supplies.

"It's been almost a year since we last received any kits for complicated caesareans. I'm not quite sure why, but, as I understand it, the DRS [Regional Health Authority] has run up quite a debt on these items. To be perfectly frank, I think free healthcare has had its day. The two remaining kits, for the operation and post-operation stages, have less and less in them, and there's always something missing from them. The number of kits sent to us by the DRS is also declining. It fell from 300 to 200, and now it's only 100," (Staff member, Regional hospital, Sikasso).

"The State is no longer able to pay the PPM the kind of money that would guarantee a proper supply of products. The point is that the PPM is a business. The situation is catastrophic, and this is why our needs are fulfilled less and less. Kits for complicated caesareans have become increasingly scarce," (Official, Regional health authority, Sikasso).

At the national level, the administration can no longer guarantee reimbursement of the money spent by health centres.

"As an accountant for the referral health centre [I can tell you], if you take caesareans, we haven't had the money for 2009, let alone 2010. The State really isn't paying up," (Staff member, CSREF, Bamako).

The follow-up system, geared towards compiling the information required for reimbursement of caesareans performed, did not track how the kits were used or what inputs were wasted. The absence of any specific supervision only encouraged the liberties taken at the operational level. These conditions appeared to put the policy's sustainability at risk.

In the health centres, staff exploited the policy's complex terms of reference and product shortages, resulting in gradual erosion of the free healthcare package, with significant local discrepancies. Hence, since 2011, there has been clear evidence of a decline in the quality of services and a gradual shrinking of the scope of free healthcare, with the writing of prescriptions (obliging users to buy their own products) becoming almost a matter of course for each caesarean.

### The policy of free healthcare for malaria

#### National policy background

User fee exemption for the treatment of malaria in children under five and pregnant women is Mali's most recent policy, introduced by the government in 2007. It is particularly ambitious, in that it involves all the country's health centres and targets their two main clienteles: pregnant women and children under five. In fact, malaria is the primary reason for consultation in health centres. The free healthcare package consists of preventive treatment (sulfadoxine/pyrimethamine) for pregnant women, insecticide-treated nets, treatment of both uncomplicated and severe malaria, and rapid diagnostic testing (RDT) for under-fives and pregnant women. To qualify for free healthcare, the malaria diagnosis must be confirmed by RDT or thick blood smear.

This policy is being steered by the PNLP (national malaria programme) and implemented by decentralized services reporting directly to the National Directorate of the Ministry of Health. The policy enjoys substantial financial support from a variety of sources outside the country, including the Global Fund, the (US) President's Malaria Initiative (PMI), and the World Bank, among others.

#### The implementation of this policy in healthcare facilities

Officially, health professionals have not questioned the policy. The presidential measures are difficult to fault and, in terms of public health, this measure is beyond reproach from an ethical standpoint. Rather, doubts about the policy are expressed by the practices of the caregivers, who clearly seek to reduce its scope. This was clearly demonstrated by the situations we observed.

Healthcare personnel are not very forthcoming with patients about free healthcare. Those who qualify for it are not told about it from that quarter, and users systematically arrive with money in their pockets, unsure of the truth of what they may have heard on the radio or TV. Moreover, at some centres, it was only during our survey that the information was communicated to users or even to community officials. It is difficult for patients to claim entitlement to a free service under such conditions.

Staff often refuse, moreover, to use RDTs or even thick blood smears to confirm clinical diagnoses. Three reasons are proffered to explain their relative indifference to RDTs: clinical evidence of malaria, frequent shortages of RDTs, and their presumed unreliability.

"Personally, I don't wait for the result of the thick blood smear to write out the prescription. We are tired of this free healthcare. The test is slow, the patient can't wait and often it's not even reliable. Fever, vomiting, diarrhoea and loss of appetite are sufficient evidence that it's malaria," (Staff member, CSCOM, Bamako).

"In any case, these tests are no good for Africans. These products are sensitive. The conditions in which they are transported, stored and even handled can cause them to give incorrect results. And this is precisely what happens, they are always giving wrong results. When a child is brought to us with all the symptoms, we do the test and it's negative. But when we prescribe anti-malarial drugs, we see him get better," (Staff member, CSCOM, Sikasso).

Lastly, the availability of free inputs in health centres is very hit and miss. Although the blame for this rests partly with the central administration, which is faced with a daunting challenge, operational level practices also bear considerable responsibility. They are clearly aimed at limiting access to free treatment and at reducing the free healthcare package. Most health centres submit reports detailing their needs late and at irregular intervals, which impedes planning along the whole chain (district, DRS and PPM). In Bamako, reporting is particularly lax, and this has gradually become accepted.

"From the start of free healthcare in 2007 until now, we've never kept stock records. In any case, I don't have the time for that,... we've got enough work as it is. What's free is free. I make a request as soon as stock is low or has run out, or I may even phone the medical officer and inform him of the situation, and he sends me something," (Staff member, CSCOM, Bamako).

In the regions, where the situation is slightly better than in Bamako, reporting remains poor. The data for the district of Sikasso showed that fewer than 50% of the district's health centres submitted monthly reports.

In addition, shortages of inputs or incomplete test kits are not alleviated by borrowing from the stock of local health centres pending the next delivery. The governing body of the community health associations even advises its members, who manage the community health centres, to charge users whenever stock shortages occur, citing the maxim: "If there are no free medicines, there is no free treatment for users."

Finally, even when stocks of free inputs are available in health centres, it does not necessarily follow that they are accessible to the targeted categories of users. Staff take advantage of certain loopholes in the system to engineer local shortages: health centres are slow to collect their allocations depending on local travel opportunities. It is not uncommon for stock to be available at the referral health centre while shortages of free inputs are announced in outlying areas. At the same time, free inputs are often less closely guarded than those that generate revenue and hence are subject to all kinds of temptations, especially theft. Staff may also set aside a box of free products without using them so that, if there is an inspection, they are not taken to task for allowing supplies to run out:

"We haven't had Co-Artesiane in stock for about ten days, apart from a box that we are hanging on to, as we could be inspected and caught without stock, which doesn't look good," (Staff member, CSREF, Kita).

'Oversights' in the prescribing of medicines also occur, raising the question of whether such lapses might be due to the desire to limit free healthcare; some respondents acknowledged this was the case.

"The kits are not brought out, the doctors tell me they forget to prescribe the kit for pregnant women. They let all severe malaria cases pay for their own prescriptions. The reality is that we are looking for money here at the community health centre," (Staff member, CSCOM, Bamako).

Finally, access to free treatment was restricted to workdays and working hours, i.e., between 8 am and noon or 2 pm, the managers having decided that staff on duty outside these times, usually junior staff or trainees, were not sufficiently reliable to manage free inputs.

The policy has not been backed up by funding for supervision, which is therefore incorporated into the general supervision routinely conducted by district teams. Between 2009 and 2011, this routine supervision was rarely carried out due to lack of funding. Hence it can be difficult to identify problematic practices at the local level.

"We make do with the reports sent in by centre heads. No one knows whether the products we give them are put to proper use. In Mali, everyone wants to protect their job and no one dares to say what things are really like," (Official, Regional health authority, Bamako).

Healthcare is not entirely free, as consultations and medicines other than anti-malarials have to be paid for (antipyretics and antibiotics). In the community health centres observed in our study, we compared, for the months of January and July of the study year, the average monthly bills for prescriptions for uncomplicated or severe malaria, both with and without free healthcare. The evidence showed that, despite the free medicines policy, the amounts users are required to pay remain substantial and prohibitive for disadvantaged families. For instance, in one CSCOM in Bamako, under the free healthcare system the average cost of a prescription for treating an uncomplicated case of malaria was XOF 2,280 and for a severe case, XOF 4,337-only about XOF 1,000 and 1,500 less, respectively, than for cases that did not qualify for free treatment (for example, children five years and older).

The informal practices of health professionals reduced the scope of the policy further still. Under such conditions, free healthcare has little meaning, and thus it is hardly surprising that its impact would be hampered. In fact, a study on the impact of free healthcare on visits to health centres in the different survey areas [18] confirmed that, although there had been a real increase in the number of visits, the impact remained limited.

## Discussion

### Comparison of these policies reveals systematic malfunctioning in the health system

In presenting and comparing these three policies, whose provisions and implementation conditions were all different, this article shows that while they have all had generally positive outcomes in terms of quantitative impacts on health centre use, they are being progressively stripped of any real content, and their sustainability is uncertain precisely because of the malfunctions of the system in which they are being implemented. While the national level has had problems with planning, even more problematic has been its incapacity to supervise and control health workers' illicit practices to compensate for salaries they consider insufficient and which have not been affected by the policies. In such a context, free care policies are like a grain of sand in clockworks, an irritant in the health centres' parallel functioning that incites workers to implement strategies that undermine the policies' sustainability. Thus, contrary to the expectations often expressed, free healthcare policies, as implemented in this context, do not constitute an opportunity to structure the health system more effectively [19].

These policies were thus adopted differently by health professionals, and their acceptability varied depending on the extent to which they disrupted the habitual functioning of the health system.

The concern for optimising HIV/AIDS management led to implementation of a mechanism that was technically and financially supported by international donors but not well integrated into the health system. This came at a price, which was heavy dependence on external funding and progressive development of a two-tiered system, public vs. associative, in which the public sector had no share in the advantages made available to the associative sector by the Global Fund, such as staff bonuses. In public healthcare facilities, initial acceptance of this policy and of some promising and effective innovations (psychosocial approaches, adherence clubs, careful planning) was gradually replaced by a commoditisation of treatment rather than by an extension to other pathologies of the principles underlying its success.

The free caesareans policy, whose measures were fully integrated into healthcare services, was gradually absorbed into the system. The discontent expressed about increased workload without financial compensation in fact concealed other demands that could not be articulated because they referred to dysfunctional practices that could not be openly revealed: the absenteeism of certain categories of personnel; the use of volunteer or underpaid staff, some of whom kept the services running for several consecutive years; and the loss of informal income that had, until then, significantly supplemented the incomes of certain categories of personnel, particularly managers. As well, the wastage of caesarean kits because of lack of supervision led to a progressive undermining of the free healthcare package and a substantial decline in quality of care. Thus, a recent study has shown that the mean cost of treatment is not significantly different between women receiving free caesareans (USD 107.00) and those with normal deliveries (USD 93.40). As such, in this case, free healthcare no longer protects the beneficiaries from catastrophic expenditure [14].

Free malaria treatment for children under five and pregnant women, an ambitious and complex policy, is a disruptive policy that provokes latent but effective opposition. In a context where, according to the health authorities and the managers of the health centres studied, the sale of anti-malarials is the major source of income for health centres, these free inputs provided to healthcare facilities and the requirement for clinical diagnoses to be confirmed through biological testing to limit unwarranted malaria diagnoses arouse considerable anxiety among both health professionals and the community leaders of CSCOMs. This anxiety, motivated by this policy's ostensibly negative impact, has no basis in reality, as demonstrated by the results of a study that very clearly showed there was no such financial impact in any of the CSCOMs studied [20]. In fact, because of the failure to consider beforehand that the user fee exemption for malaria would threaten the substantial income stream that malaria represented for healthcare facilities, this free policy is now faltering everywhere in Mali, including in the sentinel sites, which are theoretically the best-performing facilities.

The conclusion that health services malfunctions impede free care policies, which in turn hamper the strategies implemented by health professionals, was reached for other countries by our research program studying largely similar health systems [21]. Our conclusions also agree with those of a literature review on the weaknesses and malfunctions either produced or exacerbated by exemption polices [11], whose consequences included, in most cases, a decline in the quality of care.

The findings of this study, revealing malfunctions ranging all the way from the centre to the most peripheral points of the health system, raise questions about how much attention is really given to the stated objective of these policies, i.e., improving the population's access to care.

The idea that this issue of the financial accessibility of services is not a core concern for health professionals is corroborated by the findings of other studies as well. The cost-recovery policy has been accepted everywhere and applied zealously, and a study exploring health professionals' views on this policy showed it was widely appreciated despite its disastrous consequences on access to care [22]. Moreover, the failure of the user fee exemption for indigents that had been envisioned in the Bamako Initiative [23] is another illustration of the futility of attempts to reduce inequities of access to care.

With the advent of structural adjustments, the State's disengagement (reduced public funding) foisted onto healthcare facilities, and particularly onto CSCOMs and hospitals, an obligation to achieve results and balance budgets-i.e., to be more efficient. This has since clearly evolved into a quest for profitability, whether individual or collective.

We have highlighted the personal profit motives underlying the health professionals' actions (profits generated by their illicit practices, which run counter to the interests of patients or of the health centres in which they work) and undermining the free care policies.

Thus, a study carried out across the entire region of Sikasso highlighted the healthcare facilities' solid financial status (profits generated at year's end and sometimes even financial reserves) and, at the same time, their inadequate equipment and the absence of any measures (lower fees, free care for indigents) to improve equity of access to care [24]. In centres with financial surpluses, those cash reserves and surpluses were neither re-invested in equipment for the centre, nor put into a fund to support indigents, but instead re-invested as end-of-year bonuses distributed to personnel. Another study, this time in Burkina Faso, showed that health centre management committees could viably use their financial resources to improve the equity of access to care, but that this was not something that occurred to them spontaneously [25].

As such, improving the financial conditions for users' access to good-quality services appears to be a secondary consideration.

## Conclusion

This study of the actual implementation of the three most recent user fees exemption policies in Mali shows that they significantly increased the use of services and free care and have been much appreciated by users. However, these policies potentially undermine certain practices of health professionals, whose primary concern is to improve their own remuneration, which they consider to be inadequate. As such, the scope of these policies has gradually become more limited, jeopardizing their sustainability. In countries that have functioned for the past 30 years according to a cost recovery model, user fee exemption measures are seen as a relatively abrupt step backward. Health professionals' self-interest with regard to user fees appears to have been underestimated [22]. The free healthcare policies sparked latent opposition, expressed essentially through informal professional practices. In fact, the policies cannot be contested officially as they are eminently political and ethically justified in terms of public health. This raises the question of whether there really is a shared concern to improve equity in access to care, and whether the Malian State has the capacity to impose a certain social justice, such as bringing an end to the current climate of impunity manifested in health workers' informal practices. The reactions of national authorities and international bodies to this problem have not been equal to the task: imposing successive reforms, with no provisions for appropriate evaluation despite repeated failures, rather than looking in-depth at African health systems and taking into account their structural weaknesses, and especially the profit-generating logic in which they are now entangled, to the users' detriment.

This is not a matter of designating health professionals as the new scapegoats. Rather, these malfunctions reflect the State's incapacity to exercise its regulatory role and to establish policies that are "adapted to African health systems as they are in real life, and not on paper, taking into account their dysfunctions, their difficulties, their blockages, their bottlenecks. Which means recognizing them and denouncing them" [author's translation] [26]. International development agencies, for their part, have not managed, in the 10 years since the Paris Declaration, to align all their actions and practices with the national policy framework [27], to harmonise their interventions-as evidenced very recently in the management of the crisis in northern Mali [28]-and to move away from a liberal conception that has, since the Bamako Initiative, permeated most public policies related to health. Moreover, a review of the literature on the adverse consequences of innovations has shown that studies on this topic are rare, with the main limiting factor being funding agencies' bias toward funding only studies on the intended consequences of innovations, and not unintended or negative consequences [29]. Too often, "new public policies overlook routine practices and presuppose an ideal health system-and State-far removed from the everyday reality of the health system-and of the State" [author's translation] [26].

## Competing interests

None

## Authors' contributions

LT designed, collected, processed and analysed the data and wrote the draft and final version of the manuscript. All authors read and approved the final manuscript.

## Declarations

This article has been modified from the chapters **La gratuité comme révélateur des dysfonctionnements ordinaires du système de santé au Mali. Approche comparée des exemptions de paiement concernant le sida et la césarienne** by L. Touré [30], and **Les exemptions de paiement du traitement du paludisme pour les enfants de moins de 5 ans et les femmes enceintes: une politique qui dérange** by L. Touré and Y. Sanogo [31] in the book *Une politique publique de santé et ses contradictions. La gratuité des soins au Burkina Faso, au Mali et au Niger*, J.-P. Olivier de Sardan and V. Ridde (eds), 2014, Karthala, Paris, with the permission of the publisher. The publication of this supplement was funded by a grant from International Development Research Centre (IDRC), Ottawa, Canada.

This article has been published as part of *BMC Health Services *Research Volume 15 Supplement 3, 2015: User Fee Exemption Policies. The full contents of the supplement are available online at http://www.biomedcentral.com/bmchealthservres/supplements/15/S3.
